# The influence of pediatricians' recommendation on caregivers' COVID-19 vaccine acceptance for children: A nationwide cross-sectional survey study from USA

**DOI:** 10.3389/fped.2023.1149125

**Published:** 2023-05-09

**Authors:** Pritish Mondal, Ankita Sinharoy

**Affiliations:** ^1^ Department of Pediatrics, Penn State College of Medicine, Hershey, PA, United States; ^2^ Heart and Vascular Institute, Penn State College of Medicine, Hershey, PA, United States

**Keywords:** COVID-19, vaccine hesitancy, parental vaccination decision-making, pediatrician attitudes, machine learning, artificial neural network - ANN, national survey, COVID-19 vaccine acceptance

## Abstract

**Background:**

The influence of pediatricians on parental acceptance of COVID-19 vaccine for children has not been well studied. We designed a survey to estimate the impact of pediatricians' recommendations on caregivers' vaccine acceptance while accounting for participants' socio-demographic and personal characteristics. The secondary objectives were to compare childhood vaccination rates among different age groups and categorize caregivers' concerns about vaccinating young (under-five) children. Overall, the study aimed to provide insight into potential pro-vaccination strategies that could integrate pediatricians to alleviate parental vaccine hesitancy.

**Methods:**

We conducted an online cross-sectional survey study using Redcap, in August 2022. We enquired COVID-19 vaccination status of the children in the family (≥five years). The survey questionnaire included socio-demographic and personal characteristics: age, race, sex, education, financial status, residence, healthcare worker, COVID-19 vaccination status and side effects, children's influenza vaccination status, and pediatricians' recommendations (1–5 scale). Logistic regression and neural network models were used to estimate the influence of socio-demographic determinants on children's vaccine status and build predictors' ranking.

**Results:**

The participants (*N* = 2,622) were predominantly white, female, middle-class, and vaccinated against COVID-19 (89%). The logistic regression model was significant vs. the null (likelihood-ratio *χ*^2^ = 514.57, *p* < 0.001, pseudo-R^2 ^= .440). The neural network model also demonstrated strong prediction ability with a correct prediction rates of 82.9% and 81.9% for the training and testing models, respectively. Both models identified pediatricians' recommendations, self-COVID-19 vaccination status, and post-vaccination side effects as dominant predictors of caregivers' vaccine acceptance. Among the pediatricians, 70.48% discussed and had an affirmative opinion about COVID-19 vaccine for children. Vaccine acceptance was lower for children aged 5–8 years compared to older age groups (9–12 and 13–18 years), and acceptance varied significantly among the three cohorts of children (*χ*^2^ = 65.62, *p* < 0.001). About half of the participants were concerned about inadequate availability of vaccine safety information for under-five children.

**Conclusions:**

Pediatricians' affirmative recommendation was significantly associated with caregivers' COVID-19 vaccine acceptance for children while accounting for participants' socio-demographic characteristics. Notably, vaccine acceptance was lower among younger compared to older children, and caregivers' uncertainty about vaccine safety for under-five children was prevalent. Thus, pro-vaccination strategies might incorporate pediatricians to alleviate parental concerns and optimize poor vaccination rate among under-five children.

## Introduction

Centers for Disease Control (CDC) recommends vaccinating children against COVID-19 since it is safe and effectively prevents severe COVID-related complications (1). During the early days of the pandemic, COVID-19 was perceived as a disease primarily affecting older adults (2). However, in late 2021 the Omicron wave came to the USA, and both incidence and hospitalization among children increased by several folds compared to the earlier spikes (3). By the end of April 2023, 15.5 million children tested positive for COVID-19 in the USA (4), while around 17,400 COVID-related deaths were reported globally (by March 2023) among pediatric population (5). Several countries, including the USA, have relaxed mandatory preventive practices as we try to return to the pre-pandemic lifestyle. However, in April 2023, 48,000 new pediatric COVID-19 cases were reported in the USA (4), indicating that COVID-19 is still a major public health concern. While children usually experience less severe disease compared to adults, children with immunodeficiency, chronic conditions, obesity, and asthma are certainly at a higher risk for developing significant post-COVID complications, especially if unvaccinated (6). Importantly, if a substantial number of children receive COVID-19 vaccine, it would help to build herd immunity (7).

Parental vaccine hesitancy is multi-factorial. While various socioeconomic characteristics have been reported to influence caregivers' COVID-19 vaccine acceptance for children, such studies rarely acknowledged pediatricians' opinions as a potential determinant of parental decision (8). However, previous reports indicated the positive influence of pediatricians on parental, especially mothers’ decision to immunize their children (9–11). On May 2021, US Food and Drug Administration (FDA) first authorized the COVID-19 vaccine for adolescents and subsequently for children above the age of five (12). About half of the children (52.9%) aged five years or above have been vaccinated by the beginning of April 2023 (13). In June 2022, FDA authorized COVID-vaccine for children aged between six months to five years (14), and to date, the rate of vaccination has been only around 11% for under-five children (15). Among various socio-demographic factors that lead to parental vaccine hesitancy, the influence of age, race, sex, level of education, family income, and state of residence has been well described (16–18). Recent studies from the USA and other countries also suggested that parental knowledge significantly influenced children's vaccine acceptance (19, 20).

Parents are usually concerned about the potential side effects of COVID-19 vaccine irrespective of their background and often believe that immunizing children may not be the best decision, considering the risk-to-benefit ratio (21). Pediatricians can encourage and educate parents to alleviate vaccine hesitancy, especially if they are indecisive, and may help to improve the overall rate of immunization (22–25). In addition, parents often trust pediatricians more than official reports and guidelines, which could be utilized as a public health strategy. However, knowledge of pediatricians' influence on parental COVID-19 vaccine acceptance is vastly underreported and might help us to build a different approach to improve vaccination coverage. To address the knowledge gap, we conducted a cross-sectional survey to recognize the key determinants of caregivers' decision to vaccinate household children (five years or above) against COVID-19. We hypothesized that “Pediatricians” strong recommendations would influence parents to overcome vaccine hesitancy against COVID-19'. Therefore, a survey was designed to determine the impact of pediatricians' recommendations on caregivers' decisions compared to participants' socio-demographic and other personal characteristics. The secondary objectives were comparing the rate of COVID-19 vaccination among children of different age groups and categorizing parental concerns about vaccinating young (under-five) children.

## Materials and methods

### Study design, settings, and ethics

We conducted an online cross-sectional survey between 11th to 30th of August 2022. The survey questionnaire was built using Redcap (Supplementary Table S1). Penn State Institutional Review Board approved the study protocol. Please review Supplementary File S2 for the details about pilot study and preparatory phase.

### Participant recruitment

We used an NIH-funded non-profit web-based portal “ResearchMatch” to recruit survey participants (26). ResearchMatch offers access to adult volunteers of all diversities, including ethnicity, age, race, sex, and US states. The survey inclusion criteria were the age of 18 years or above and US residency. This was an unpaid survey. Implied consents were obtained at the beginning of the survey, and the responses were stored in the Redcap database.

### Survey instruments and characterization of socio-demographic determinants

We collected data on the participants' socio-demographic characteristics, such as age, race, sex, level of education, family income, and occupation (Table 1). Survey participants were characterized based on their age (five groups in ascending order), race (eight categories), sex (three categories), level of education (five levels from “high school” to “PhD.” in ascending order), family income (four categories in ascending order), and healthcare worker (yes vs. no). The participants' residence states were also regrouped into ten US Health and Human Services (HHS) regions (27). We asked whether the study subjects have children aged between 5 and 18 years in their families. Children were stratified into three subgroups based on their age: 5–8 years, 9–12 years, and 13–18 years. We also documented the COVID-19 and Flu vaccination status (Yes vs. No) of survey participants and family children. Finally, post-vaccination side effects experienced by the participants and their children were classified and quantified on a 0–10 severity Likert scale (Table 2). SPSS 28 and SAS 9.4 were used for data analysis.

**Table 1:** Socio-demographic and other characteristics of survey participants.

Socio-demographic characteristics	Subgroups	*N* (%)
Age (score 1–5 in ascending order) (*N* = 2,601)	Level 1 (18–24 years)	104 (4%)
	Level 2 (>24–44 years)	907 (34.9%)
	Level 3 (>44–60 years)	632 (24.3%)
	Level 4 (>60–70 years)	532 (20.5%)
	Level 5 (>70 years)	426 (16.4%)
Sex (*N* = 2,600)^a^	Male	611 (23.5%)
	Female	1,973 (75.9%)
	Others	16 (0.6%)
Pediatricians’ recommendation score (*N* = 928)	1	22 (2.37%)
	2	82 (8.84%)
	3	170 (18.32%)
	4	246 (26.51%)
	5	408 (43.97%)
Race/Ethnicity^a^ (*N* = 2,598)	Black	124 (4.8%)
	Hispanic/Latino	98 (3.8%)
	White	2,196 (84.5%)
	Asian	39 (1.5%)
	Asian Indian	26 (1%)
	Belongs to two or more races	58 (2.2%)
	Middle Eastern	14 (0.5%)
	Others	43 (1.7%)
Healthcare worker^a^ (*N* = 2,585)	Yes	606 (23.4%)
	No	1,979 (76.6%)
Education (Score 1–5 in ascending order) (*N* = 2,608)	Level 1 (High School or less and others)	118 (4.5%)
	Level 2 (Undergraduate/some college)	648 (24.7%)
	Level 3 (Graduate)	733 (28%)
	Level 4 (Masters)	693 (26.4%)
	Level 5 (Doctorate/Professional degree (MD, MBA, LAWYER etc..)	416 (15.9%)
Financial status (Score 1–5 in ascending order) (*N* = 2,593)	Level 1 (Lower middle class/poor)	332 (12.8%)
	Level 2 (Middle class)	1,457 (56.2%)
	Level 3 (Upper middle class)	753 (29.0%)
	Level 4 (Wealthy)	51 (2.0%)
Participants’ COVID vaccination status^a^ (*N* = 2,583)	Yes	2,299 (89%)
	No	284 (11%)
Participants’ COVID vaccination status^a^ (*N* = 2,595)	Yes	2,147 (82.7%)
	No	448 (17.3%)
Child's Influenza vaccination status^a^ (*N* = 1,035)	Yes	779 (75.27%)
	No	256 (24.73%)
HHS category^a^ (*N* = 2,595)	Region 1 (Boston)	77 (3.0%)
	Region 2 (New York)	232 (8.9%)
	Region 3 (Philadelphia)	274 (10.6%)
	Region 4 (Atlanta)	652 (25.1%)
	Region 5 (Chicago)	575 (22.2%)
	Region 6 (Dallas)	174 (6.7%)
	Region 7 (Kansas City)	90 (3.5%)
	Region 8 (Denver)	118 (4.5%)
	Region 9 (San Francisco)	261 (10.1%)
	Region 10 (Seattle)	142 (5.5%)

The number of participants who responded to Pediatricians’ recommendation score and child's Flu vaccination status was significantly less than the number of completed responses since all the participants did not have children or knew vaccination status of family children.

^a^ Categorical variables.

**Table 2:** Post-COVID vaccination side effects experienced by study participants and children in the family.

	Survey Participants	Family Children
**Post-vaccination side effects**		
Fever	34.32%	26.69%
Local pain	28.53%	19.45%
Gastrointestinal	13.96%	8.42%
Respiratory	9.73%	8.07%
Musculoskeletal	7.74%	1.30%
Fatigue	6.52%	4.98%
Other	6.25%	2.61%
Body aches & headache	3.81%	1.30%
Cardiac	3.32%	1.19%
Hematological	1.11%	0.24%
**Severity of side effects on 0–10 Likert scale**		
Mild (0–3)	71.39%	85.05%
Moderate (4–6)	19.47%	12.34%
Severe (7–10)	9.13%	2.61%

The percentage of side effects and severity (%) were calculated among the number of vaccine recipients.

### Data sources/measurement

Our data source was the survey responses captured in RedCap. Most of the answers were either in “Yes” vs. “No” dichotomous variables (for example, COVID-19 vaccine received, healthcare worker), categorical variables (for example, race and US state of residence), or on various Likert scales. We used *χ*^2^ tests to determine the difference in vaccine acceptance rates among the children of various age groups.

### Bias

The selection of the participants was a concern in terms of selection bias, since we could only access a pre-defined cohort. “Researchmatch” offers access and the opportunity to recruit study participants across the country, which was not necessarily a representative of US population demographics (Supplementary File S2). Nevertheless, the study participants' relatively uniform distribution across the country perhaps mitigated selection bias to some extent.

### Sample size estimation

Since the percentage of COVID-19 vaccine acceptance among participants was unknown, we assumed that half of the study subjects would be pro-vaccination. Considering the adult US population of 332,403,650 in early 2023 (28), a minimum of 1,068 survey participants would be required to achieve a confidence level of 95% with a margin of error of 3%, with a pre-defined acceptance rate of 50% (29).

### Quantitative variables

Pediatricians' COVID-19 vaccine recommendation: We asked about pediatricians' opinions and recommendations encouraging COVID-19 vaccination. The responses were characterized into five categories: strong recommendation, recommendation, neutral opinion, recommended against, and no discussion, respectively. We assigned scores of “5′ and “4” on a “1–5” Likert scale as strong and favorable recommendations, respectively. A score of “3” was assigned for no conversation and “2” for neutral opinion, considering pediatricians' neutral opinion could be perceived as less encouraging to the families than having no conversation. Finally, a score of “1” was designated if the pediatrician advised against the COVID-19 vaccine.

### Internal consistency

Internal consistency among the responses indicating study participants' decision to vaccinate family children of different age groups (“No” vs. “Yes” responses were captured numerically as 0 and 1), and pediatricians' recommendation (1–5 scale) to vaccinate children of three different age groups were estimated using Cronbach's alpha.

### Prediction models

We used two methods to estimate the influence of various independent variables (socio-demographic and personal characteristics, including pediatricians' opinions) on the study participants' decision to vaccinate the children in the family (Tables 3, 4). Several respondents had more than one child in the family and had diverse opinions about vaccination for different age groups. Thus, the respondents were categorized into positive, negative, and mixed vaccine acceptance categories.

**i) Multinomial logistic regression model:** We used logistic regression since the dependent variable (participants' vaccine acceptance) was categorically distributed (Tables 3, 4), while independent variables were comprised of both categorical and continuous variables. The strength of the model or goodness of fit was estimated by the likelihood ratio test (*χ*^2^, significance) and McFadden's pseudo *R*^2^. Most influential predictors were identified based on the value of “−2 Log Likelihood of Reduced Model”, or an estimation of how much the model would change if that variable was removed (30). It is also defined as model fitting criteria.
**ii) Multilayer Perceptron Neural Network (MLPNN) model:** MLPNN model has been applied extensively in healthcare-related research (31, 32). We used the MLPNN model to estimate the degree of influence of the determinants on caregivers’ COVID-19 vaccine acceptance for their children. In the first step, potential socio-demographic predictors were selected as an input layer (also known as factors), and COVID-19 vaccine acceptance scores were designated as the dependent variable. Next, the dataset was randomly partitioned, with 80% data selected as the training and 20% as the testing sample. Subsequently, the machine learning tool built the model architecture with an automatically selected number (range: 1–50) of hidden layers. Finally, the outcome layer was created by the MLPNN tool, depending on each of the hidden layers' contributions (Table 3). The output comprised a rank list based on the relative importance of the predictors. The percentage of accurate prediction in the training and testing model indicated the overall significance of the MLPNN model.

**Table 3:** Logistic regression and neural network models estimating the influence of pediatricians’ recommendation and participants’ socio-demographic characteristics and caregivers’ COVID-19 vaccine acceptance for children.

Socio-demographic Predictors	Multinomial logistic regression				MLPNN	
	−2 Log likelihood of reduced model^a^	*χ* ^2^	*p*-value	Rank	Relative importance (%)	Rank
Participants’ COVID Vaccination Status	758.753	108.96	<.001*	1	20.95%	1
Pediatricians’ Recommendation Score	758.143	108.35	<.001*	2	17.46%	2
Participants’ Post-Vaccination Side Effects	686.176	36.38	.014*	3	9.50%	3
HHS Category	680.39	30.60	.032*	4	8.14%	5
Race	673.48	23.69	.049*	5	5.37%	10
Child's Influenza Vaccination Status	668.5	18.71	<.001*	6	7.83%	6
Age	667.909	18.12	.020*	7	6.12%	8
Financial Status	659.542	9.75	0.136	8	8.70%	4
Healthcare Worker	659.02	9.23	.010*	9	3.32%	11
Gender	658.193	8.40	0.078	10	6.06%	9
Level of Education	655.356	5.56	0.696	11	6.55%	7

a Model Fitting Criteria and the rank list of the predictors in regression model was computed based on the value of “−2 Log Likelihood of Reduced Model”, which estimated the degree of change in regression model if that variable was removed from the analysis. The ranking of the predictors in MLPNN model was determined by their relative importance.

* *p*-value <0.05 was considered significant. MLPNN, multilayer perceptron neural network.

**Table 4:** Parameter estimates for logistic regression model estimating predictors of caregivers’ COVID-19 vaccine acceptance for children in the family.

Dependent variable (COVID-19 vaccine acceptance for children)^a^	Independent variables (Predictors of COVID-19 vaccine acceptance)	Categories	B	Standard error	Wald	df	*p*-value	Exp (B)	95% CI for Exp (B) (Lower bound)	95% CI for Exp (B) (Upper bound)
Negative response	Intercept		−21.378	583.099	0.001	1	0.971			
	Post-COVID vaccination side effects (0–10 scale)	0	−1.157	0.981	1.389	1	0.239	0.315	0.046	2.153
		1	−1.343	1.195	1.264	1	0.261	0.261	0.025	2.714
		2	−0.813	1.065	0.582	1	0.445	0.444	0.055	3.578
		3	−0.516	1.028	0.252	1	0.616	0.597	0.08	4.477
		4	−1.702	1.058	2.584	1	0.108	0.182	0.023	1.452
		5	−0.834	1.059	0.62	1	0.431	0.434	0.055	3.459
		6	−1.436	1.143	1.579	1	0.209	0.238	0.025	2.234
		7	0.208	1.08	0.037	1	0.847	1.231	0.148	10.234
		8	0.827	1.12	0.545	1	0.46	2.287	0.254	20.561
		9	−0.713	1.319	0.292	1	0.589	0.49	0.037	6.501
		10	0^b^			0				
	Age	Level 1	−2.053	1.659	1.531	1	0.216	0.128	0.005	3.317
		Level 2	0.317	0.633	0.251	1	0.616	1.373	0.397	4.749
		Level 3	0.213	0.632	0.113	1	0.736	1.237	0.359	4.269
		Level 4	1.028	0.728	1.993	1	0.158	2.794	0.671	11.641
		Level 5	0^b^			0				
	Level of education	Level 1	1.216	0.733	2.749	1	0.097	3.372	0.801	14.187
		Level 2	0.717	0.434	2.735	1	0.098	2.049	0.876	4.792
		Level 3	0.248	0.427	0.337	1	0.562	1.281	0.555	2.958
		Level 4	0.216	0.427	0.255	1	0.614	1.241	0.537	2.865
		Level 5	0^b^			0				
	Financial status	Level 1	14.686	583.082	0.001	1	0.98	23,88,892.92	0	.c
		Level 2	14.712	583.082	0.001	1	0.98	24,49,837.62	0	.c
		Level 3	14.443	583.082	0.001	1	0.98	18,72,577.74	0	.c
		Level 4	0^b^			0				
	Race	African-American	2.785	1.777	2.456	1	0.117	16.198	0.497	527.479
		Hispanic/Latino	2.792	1.814	2.369	1	0.124	16.311	0.466	570.735
		White	2.563	1.717	2.229	1	0.135	12.976	0.448	375.486
		Asian	−11.222	876.811	0	1	0.99	1.34×10^−05^	0	.c
		Asian Indian	−11.629	750.021	0	1	0.988	8.90×10^−06^	0	.c
		Belongs to ≥2 races	3.169	1.784	3.155	1	0.076	23.789	0.721	785.324
		Middle Eastern	2.256	2.113	1.14	1	0.286	9.54	0.152	599.554
		Others	0^b^			0				
	Healthcare worker	No	−0.468	0.288	2.637	1	0.104	0.627	0.356	1.102
		Yes	0^b^			0				
	HHS region	Region 1	2.254	0.99	5.185	1	0.023	9.524	1.369	66.276
		Region 2	0.918	0.859	1.141	1	0.285	2.504	0.465	13.488
		Region 3	−0.036	0.853	0.002	1	0.966	0.965	0.181	5.138
		Region 4	1.13	0.767	2.17	1	0.141	3.097	0.688	13.934
		Region 5	0.909	0.772	1.386	1	0.239	2.483	0.546	11.279
		Region 6	0.989	0.851	1.35	1	0.245	2.687	0.507	14.242
		Region 7	0.655	0.996	0.433	1	0.511	1.926	0.273	13.558
		Region 8	0.831	0.837	0.986	1	0.321	2.295	0.445	11.832
		Region 9	0.814	0.85	0.918	1	0.338	2.257	0.427	11.939
		Region 10	0^b^			0				
	Participant's COVID vaccination status	No	3.867	0.453	72.773	1	<.001	47.804	19.661	116.233
		Yes	0^b^			0				
	Child Influenza vaccinated	No	1.039	0.29	12.848	1	<.001	2.826	1.601	4.988
		Yes	0^b^			0				
	Pediatrician recommendation score	1	3.334	0.785	18.05	1	<.001	28.044	6.024	130.547
		2	3.757	0.474	62.889	1	<.001	42.799	16.913	108.302
		3	2.665	0.394	45.755	1	<.001	14.364	6.637	31.088
		4	1.812	0.372	23.696	1	<.001	6.12	2.951	12.691
		5	0^b^			0				
	Gender	Male	0.563	4.064	0.019	1	0.89	1.756	0.001	5,061.272
		Female	0.081	4.057	0	1	0.984	1.084	0	3,080.361
		Other	0^b^			0				
Mixed response	Intercept		−32.925	1,560.633	0	1	0.983			
	Participants post-vaccination side effects	0	13.978	1,560.63	0	1	0.993	11,75,936.267	0	.c
		1	0.076	1,660.212	0	1	1	1.078	0	.c
		2	14.091	1,560.631	0	1	0.993	13,17,390.073	0	.c
		3	14.382	1,560.63	0	1	0.993	17,62,419.862	0	.c
		4	14.643	1,560.63	0	1	0.993	22,86,893.28	0	.c
		5	12.627	1,560.63	0	1	0.994	304,623.687	0	.c
		6	13.03	1,560.631	0	1	0.993	455,928.854	0	.c
		7	13.949	1,560.63	0	1	0.993	11,43,337.767	0	.c
		8	0.776	1,743.265	0	1	1	2.172	0	.c
		9	−0.269	1,998.32	0	1	1	0.764	0	.c
		10	0^b^			0				
	Age	Level 1	−0.124	1.793	0.005	1	0.945	0.884	0.026	29.67
		Level 2	−1.002	0.8	1.568	1	0.211	0.367	0.076	1.762
		Level 3	−1.377	0.841	2.682	1	0.101	0.252	0.049	1.311
		Level 4	1.064	0.909	1.37	1	0.242	2.898	0.488	17.217
		Level 5	0^b^			0				
	Level of education	Level 1	1.044	1.199	0.758	1	0.384	2.84	0.271	29.785
		Level 2	0.414	0.782	0.28	1	0.596	1.513	0.327	7.014
		Level 3	0.178	0.757	0.056	1	0.814	1.195	0.271	5.271
		Level 4	0.307	0.769	0.159	1	0.69	1.359	0.301	6.133
		Level 5	0^b^			0				
	Financial status	Level 1	1.72	1.716	1.005	1	0.316	5.586	0.193	161.304
		Level 2	1.109	1.65	0.452	1	0.502	3.031	0.119	76.919
		Level 3	0.831	1.68	0.245	1	0.621	2.296	0.085	61.842
		Level 4	0^b^			0				
	Race	African-American	2.157	1.673	1.663	1	0.197	8.645	0.326	229.419
		Hispanic/Latino	0.123	1.96	0.004	1	0.95	1.131	0.024	52.765
		White	0.176	1.562	0.013	1	0.91	1.192	0.056	25.45
		Asian	1.045	2.446	0.182	1	0.669	2.843	0.024	343.555
		Asian Indian	0.835	2.235	0.14	1	0.709	2.305	0.029	184.271
		Belongs to ≥2 races	−12.853	786.714	0	1	0.987	2.62×10^−06^	0	.c
		Middle Eastern	2.359	2.103	1.259	1	0.262	10.584	0.172	652.329
		Others	0^b^			0				
	Healthcare worker	No	1.338	0.666	4.031	1	0.045	3.81	1.032	14.058
		Yes	0^b^			0				
	HHS region	Region 1	2.992	1.355	4.875	1	0.027	19.92	1.399	283.568
		Region 2	−0.114	1.439	0.006	1	0.937	0.892	0.053	14.985
		Region 3	−0.439	1.389	0.1	1	0.752	0.645	0.042	9.815
		Region 4	0.684	1.201	0.325	1	0.569	1.983	0.188	20.88
		Region 5	0.159	1.237	0.017	1	0.898	1.173	0.104	13.256
		Region 6	−0.214	1.43	0.022	1	0.881	0.808	0.049	13.314
		Region 7	−12.771	767.535	0	1	0.987	2.84×10^−06^	0	.c
		Region 8	1.979	1.255	2.486	1	0.115	7.233	0.618	84.603
		Region 9	0.023	1.409	0	1	0.987	1.023	0.065	16.201
		Region 10	0^b^			0				
	Participant's COVID vaccination status	No	1.959	0.692	8.004	1	0.005	7.093	1.826	27.558
		Yes	00^b^			0				
	Child Influenza vaccinated	No	1.453	0.454	10.266	1	0.001	4.278	1.758	10.408
		Yes	0^b^			0				
	Pediatrician recommendation score	1	2.451	1.11	4.88	1	0.027	11.601	1.318	102.079
		2	2.799	0.7	16.001	1	<.001	16.427	4.168	64.735
		3	1.677	0.602	7.75	1	0.005	5.348	1.643	17.415
		4	1.302	0.554	5.523	1	0.019	3.678	1.241	10.895
		5	0^b^			0				
	Gender	Male	10.603	0.767	191.10	1	0	40,272.228	8,955.705	1,81,097.10
		Female	12.107	0		1		1,81,046.803	1,81,046.803	1,81,046.80
		Other	0^b^			0				

Df, degree of freedom; CI, confidence interval.

^a^ The reference category: Positive response.

^b^ This parameter is set to zero because it is redundant.

^c^ Floating point overflow occurred while computing this statistic. Its value is therefore set to system missing.

### Caregivers’ vaccination preference

We used the *χ*^2^ test to compare vaccine acceptance among children of different age groups (Table 5). The subjects were divided into “vaccine-compliant” vs. “vaccine-hesitant” categories based on the COVID-19 vaccination status of their children. Participants with vaccine acceptance for any of the family children were considered vaccine-compliant. The *χ*^2^ tests were conducted on the aggregated data and compared the subgroups.

**Table 5:** Comparative analyses of COVID-19 vaccine acceptance among children of different age groups.

Sub Group	Vaccine acceptance *N* (Yes/No) (%)	Group-variables compared	*χ* ^2^	*p*-value
Group 1 (5–8 years) (*N* = 530)	336/194 (63.40%)	Group 1 (5–8 years) vs. Group 2 (13–18 years)	4.35	.037*
Group 2 (13–18 years) (*N* = 471)	328/143 (69.64%)	Group 1 (5–8 years) vs. Group 3 (9–12 years)	34.87	<.001*
Group 3 (9–12 years) (*N* = 541)	431/110 (79.67%)	Group 2 (13–18 years) vs. Group 3 (9–12 years)	13.50	<.001*
Participants with ≥5 children (*N* = 1,542)	1,095/447 (71.01%)	N/A		

Participants with more than one child in the family responded individually about vaccine acceptance for children of each subgroup.

*p-value <.05 was considered significant.

### Caregivers' concerns

All the participants were asked about their concerns about immunizing under-five children, irrespective of having children in the family, and the concerns or deterrents were classified into five categories such as “inadequate information about vaccine safety”, “vaccine's efficacy in disease prevention”, “children are not at risk”, “cardiac side effects”, and “vaccination is unnecessary since the child had COVID-19′.

## Results

### Socio-demographic characteristics of survey participants

We received 2,771 survey responses. Among them, 2,622 participants completed the survey and were included in the study analysis. A considerable number of respondents (44.1%) had one or more children aged five years or above. Survey participants were often mothers (54.23%) or grandparents (29.26%), and less frequently fathers (11.46%) of the child. Participants were predominantly aged between 24 and 44 years (34.9%), white (84.5%), female (75.9%), holding a graduate (28%) or master's degree (26.4%), financially belonging to the middle class (56.2%), non-healthcare worker (76.6%), and residing in HHS Region 4 Atlanta (25.1%) or Region 5 Chicago (22.2%) (Table 1). The comparison between the demographics of study participants and US national trend is described in details in Supplementary File S2 (data sheet 4).

### Pediatricians' recommendation

More than 70% of the participants indicated that the pediatrician discussed vaccinating children against COVID-19 and had an affirmative opinion (score of 4 or 5 on 1–5 Likert scale). Pediatricians’ recommendation score (mean ± SD) for the youngest age group (5–8 years) was 3.06 ± 1.90, lower compared to the score for 9–12 years (3.35 ± 1.83) and 13–18 years (3.32 ± 1.82) age groups.

### Internal consistency

Survey participants' decision to vaccinate multiple family children of different age groups demonstrated significant internal consistency (Cronbach's alpha = 0.894). Likewise, individual pediatricians' recommendations were consistent for children of various age groups who belonged to the same caregiver (Cronbach's alpha = 0.964).

### Determinants of COVID-19 vaccine acceptance for children (prediction models)

Logistic regression analysis demonstrated that the model was significant vs. the null (likelihood-ratio *χ*^2 ^= 514.57, *p* < .001, pseudo *R*^2 ^= .440). Both the models (logistic regression and MLPNN) identified pediatricians' recommendations and participants' COVID-19 vaccination status, closely followed by the side effect experienced, as the most influential predictors of vaccine acceptance for children (Table 3). Several other predictors also showed statistically significant impact, (Table 3). Parameter estimates are displayed in Table 4. MLPNN model also demonstrated significant prediction power with correct prediction rates of 82.9% and 81.9% for the training and testing models, respectively. (Details of input, hidden, and output layers of MLPNN are demonstrated in Supplementary File S3).

### Side effects of COVID-19 vaccination

About 28% of the adults (survey participants), compared to 15% of children, reported moderate (3–6 on a “1–10” scale) or severe side effects (7–10 on “0–10” severity scale) following COVID-19 vaccination. Both adults and children reported fever as the most common side effect, followed by local pain/irritation and gastrointestinal upset. However, children less frequently experienced side effects than adults (Table 2).

### Caregivers’ vaccination preference

Most of the study subjects received the COVID-19 vaccine (87.7%) and the influenza vaccine (82.7%), while COVID-19 and flu vaccine coverages for family children were 71.01% and 75.27%, respectively. COVID-19 vaccine acceptance was significantly lower (*χ*^2^ test: *p*-values <0.05) for children aged 5–8 years (63.40%) than in the older age groups (69.64% and 79.67% for 9–12 years and 13–18 years old cohort, respectively) (Table 5).

### Caregivers' concerns

We asked about potential concerns about vaccinating under-five children. Half of the participants (49.6%) expressed concern about inadequate information about vaccine safety, while 18.2% had concerns about the vaccine's efficacy in disease prevention**.** Lesser number of participants chose other reasons such as “children are not at risk” (15.79%), potential cardiovascular side effects of vaccine (12.51%), and children already had COVID-19 (7.74%) as a deterrent against vaccinating under-five children. In addition, one-third of the families (34.6%) with multiple adult members disagreed with themselves about their decision to vaccinate children.

## Discussion

Our study, based on two prediction models, demonstrated that compared to other socio-demographic factors, pediatricians' pro-vaccination recommendations significantly impacted families' decision to immunize their children against COVID-19. This underscores the critical role that healthcare providers could play in promoting COVID-19 vaccination in children, which has not been quantified in previous studies.

It's interesting to note that vaccine hesitancy was higher among caregivers of younger compared to older children. Recent CDC data (November 2022) indicated 39 coverage among children aged 5–11 years and 68% coverage in children aged 12 years and above (13). We found relatively higher vaccination rates among children aged 5–12 (66%) as well as 13–18 years (79%). Yet, by the beginning of April 2023, only 12% of under-five children have received the COVID-19 vaccine (13). Perhaps the parents of younger children have more concerns about the safety and efficacy of the COVID-19 vaccine, especially given that this age group has only recently become eligible for vaccination. The concurrent ongoing RSV and rhino-enterovirus epidemic (33, 34) and the recent downtrend of the pandemic could be affecting COVID-19 immunization rates too.

Recent data showed that one in every 30,000 COVID-19 vaccine recipients, particularly adolescents, and young adults, reported myocarditis, especially after the second dose (35). Children usually experience mild symptoms like fever and myalgia (36, 37). Despite reassuring reports, parents may still be apprehensive about potential adverse effects, which continue to challenge the COVID-19 vaccination drive in children (38–43). In 2019, the World Health Organization (WHO) reported vaccine hesitancy as a major threat to global public health, and that observation was further substantiated during this pandemic (44). About half of our survey participants expressed concern about their lack of knowledge and uncertainties around the potential side effects of the COVID-19 vaccine, and one-third of them reported intra-family disagreement about vaccinating their children. Our study further demonstrated that the participants with worse side effects were less likely to immunize family children. It is worth noting that parents often trust pediatricians more than official guidelines (45) and pediatricians' recommendations could positively influence vaccine uptake among young children (25, 38). We found that pediatricians' affirmative opinions could help to alleviate caregivers' vaccine hesitancy. Moving forward, it will be important for policymakers to incorporate pediatricians' active participation into evidence-based, culturally appropriate counseling strategies in order to improve parental vaccine acceptance. This can be done in conjunction with existing state and national-level vaccine advocacy programs, and may help to address the concerns and uncertainties that many parents have regarding COVID-19 vaccines (46).

An individual's decision to vaccinate children against COVID-19 perhaps reflects his general attitude toward immunization (16). We found that the participants who had vaccinated themselves against COVID-19 and had their children immunized against influenza were more likely to vaccinate children against COVID-19. Additionally, our study showed that the region where individuals lived (HHS category) may have significantly influenced their acceptance of vaccines. This suggests that there may be differences in vaccine attitudes and behaviors across different geographic regions of the USA.

Our study has several limitations. First, study participants were consisted of well-educated individuals with internet access, indicating convenience sampling, instead of true representation of the diversity of the US population. Second, the study participants were predominantly female and white, which reproduced previous reports of lesser interest in medical research among males and African-Americans (47, 48). Lastly, the study lacked an external cohort to validate the results. Nonetheless, our study has a few strengths. First, the results supported our working hypothesis: ‘Pediatricians’ strong recommendations influenced parents to overcome vaccine hesitancy against COVID-19' and based on the study results we infer that future pro-vaccination strategies should integrate pediatricians (49). A large sample size representing all fifty US states and enrollment of participants within a short timeframe should also be considered a strength. Finally, we used two different models identifying similar factors as primary determinants of children's vaccination status, citing the reliability of the study results. The MLPNN models also had high accuracy and consistency between training and testing samples.

In conclusion, this US-based cross-sectional survey study demonstrated that pediatricians' affirmative recommendation significantly influenced caregivers' COVID-19 vaccine acceptance for their children, regardless of the influence of other socio-demographic determinants. Our study further indicated that vaccine hesitancy was higher for younger than older children, and many survey participants expressed concerns about inadequate information on the safety of the COVID-19 vaccine. In the future, pro-vaccination campaigns should consider integrating pediatricians to alleviate parental concerns and optimize suboptimal vaccination rates, especially among under-five children.

## Data Availability

The datasets presented in this study can be found in online repositories. The names of the repository/repositories and accession number(s) can be found below: https://data.mendeley.com/datasets/5ch7f46dy6.

## References

[1] https://www.cdc.gov/vaccines/covid-19/planning/children/6-things-to-know.html#:~:text=Getting%20vaccinated%20can%20help%20protect,they%20do%20get%20COVID%2D19.

[2] Mueller AL McNamara MS Sinclair DA. Why does COVID-19 disproportionately affect older people? Aging (Albany NY). (2020) 12(10):9959. 10.18632/aging.103344 32470948 PMC7288963

[3] Steve Schering SW. CDC study: pediatric COVID-related hospitalizations rose during omicron surge American academy of pediatrics (2022). Available at: https://publications.aap.org/aapnews/news/19569?autologincheck=redirected?nfToken=00000000-0000-0000-0000-000000000000 (Accessed February 15, 2022).

[4] https://www.aap.org/en/pages/2019-novel-coronavirus-covid-19-infections/children-and-covid-19-state-level-data-report/#:~:text=As%20of%20December%201st%2C%20over,COVID%2D19%20cases%20were%20reported.

[5] https://data.unicef.org/topic/child-survival/covid-19/.

[6] Woodruff RC Campbell AP Taylor CA Chai SJ Kawasaki B Meek J Risk factors for severe COVID-19 in children. Pediatrics. (2022) 149(1). PMID: 34935038 PMCID: PMC9213563. 10.1542/peds.2021-053418 34935038 PMC9213563

[7] https://www.cnbc.com/2021/09/30/when-kids-ages-5-11-can-get-covid-vaccine-effect-on-herd-immunity.html.

[8] Miliordos K Giannouchos T Steletou E Sanidas G Karkania A Vervenioti A Parental attitudes towards vaccination against COVID-19 of children 5–11 years old in Greece. J Eval Clin Pract. 10.1111/jep.13701 35599609 PMC9347632

[9] Benin AL Wisler-Scher DJ Colson E Shapiro ED Holmboe ES. Qualitative analysis of mothers’ decision-making about vaccines for infants: the importance of trust. Pediatrics. (2006) 117(5):1532–41. 10.1542/peds.2005-1728 16651306

[10] Hough-Telford C Kimberlin DW Aban I Hitchcock WP Almquist J Kratz R Vaccine delays, refusals, and patient dismissals: a survey of pediatricians. Pediatrics. (2016) 138(3). PMID: 27573091. 10.1542/peds.2016-2127 27573091

[11] Kundi M Obermeier P Helfert S Oubari H Fitzinger S Yun J A The impact of the parent-physician relationship on parental vaccine safety perceptions. Curr Drug Saf. (2015) 10(1):16–22. 10.2174/157488631001150407104320 25859670

[12] https://www.fda.gov/news-events/press-announcements/coronavirus-covid-19-update-fda-authorizes-pfizer-biontech-covid-19-vaccine-emergency-use.

[13] https://aap.org/en/pages/2019-novel-coronavirus-covid-19-infections/children-and-covid-19-vaccination-trends/.

[14] Coronavirus (COVID-19) update: FDA authorizes moderna and Pfizer-BioNTech COVID-19 vaccines for children down to 6 months of age (2022). Available at: https://www.fda.gov/news-events/press-announcements/coronavirus-covid-19-update-fda-authorizes-moderna-and-pfizer-biontech-covid-19-vaccines-children (Accessed June 17, 2022).

[15] https://www.washingtonpost.com/health/2022/09/18/kids-coronavirus-vaccine/.

[16] Mondal P Sinharoy A Su L. Sociodemographic predictors of COVID-19 vaccine acceptance: a nationwide US-based survey study. Public Health. (2021) 198:252–9. 10.1016/j.puhe.2021.07.028 34492505 PMC8318686

[17] Alsuwaidi AR Elbarazi I Al-Hamad S Aldhaheri R Sheek-Hussein M Narchi H. Vaccine hesitancy and its determinants among arab parents: a cross-sectional survey in the United Arab Emirates. Hum Vaccin Immunother. (2020) 16(12):3163–9. 10.1080/21645515.2020.1753439 32401612 PMC8641603

[18] Oluwatosin O. Socio-demographic factors associated with childhood immunization uptake in akinyele local government area, oyo state, Nigeria. Afr J Med Med Sci. (2012) 41(2):161–7. PMID: 23185914. 23185914

[19] Fisher CB Gray A Sheck I. COVID-19 pediatric vaccine hesitancy among racially diverse parents in the United States. Vaccines (Basel). (2021) 10(1):31. 10.3390/vaccines10010031 35062692 PMC8778198

[20] Al-Qerem W Al Bawab AQ Hammad A Jaber T Khdair SI Kalloush H Parents’ attitudes, knowledge and practice towards vaccinating their children against COVID-19: a cross-sectional study. Hum Vaccin Immunother. (2022) 18(5):2044257. 10.1080/21645515.2022.2044257 35240943 PMC9225601

[21] Ruggiero KM Wong J Sweeney CF Avola A Auger A Macaluso M Parents’ intentions to vaccinate their children against COVID-19. J Pediatr Health Care. (2021) 35(5):509–17. 10.1016/j.pedhc.2021.04.005 34217553 PMC8245313

[22] Smith PJ Kennedy AM Wooten K Gust DA Pickering LK. Association between health care providers’ influence on parents who have concerns about vaccine safety and vaccination coverage. Pediatrics. (2006) 118(5):e1287–92. 10.1542/peds.2006-0923 17079529

[23] Salmon DA Pan WK Omer SB Navar AM Orenstein W Marcuse EK Vaccine knowledge and practices of primary care providers of exempt vs. vaccinated children. Hum Vaccin. (2008) 4(4):286–91. 10.4161/hv.4.4.5752 18424918 PMC5833987

[24] Gust DA Darling N Kennedy A Schwartz B. Parents with doubts about vaccines: which vaccines and reasons why. Pediatrics. (2008) 122(4):718–25. 10.1542/peds.2007-0538 18829793

[25] Dempsey AF O’Leary ST. Human papillomavirus vaccination: narrative review of studies on how providers’ vaccine communication affects attitudes and uptake. Acad Pediatr. (2018) 18(2):S23–7. 10.1016/j.acap.2017.09.001 29502633

[26] https://www.researchmatch.org/.

[27] https://www.hhs.gov/about/agencies/iea/regional-offices/index.html.

[28] https://www.commerce.gov/news/blog/2022/01/us-population-estimated-332403650-jan-1-2022.

[29] https://www.calculator.net/sample-size-calculator.html?type=1&cl=95&ci=3&pp=50&ps=332403650&x=61&y=20.

[30] https://www.ibm.com/support/pages/how-can-i-assess-variable-importance-logistic-regression-how-do-i-obtain-likelihood-ratio-test-binary-logistic.

[31] Abdar M Yen NY Hung JC-S. Improving the diagnosis of liver disease using multilayer perceptron neural network and boosted decision trees. J Med Biol Eng. (2018) 38(6):953–65. 10.1007/s40846-017-0360-z

[32] Naraei P Abhari A Sadeghian A. editors Application of multilayer perceptron neural networks and support vector machines in classification of healthcare data. 2016 Future technologies conference (FTC) (2016). IEEE.

[33] https://www.cdc.gov/mmwr/volumes/71/wr/mm7140e1.htm.

[34] https://www.theguardian.com/us-news/2022/dec/01/us-surge-respiratory-virus-rsv-influenza-children-hospitalized.

[35] Goddard K Hanson KE Lewis N Weintraub E Fireman B Klein NP. Incidence of myocarditis/pericarditis following mRNA COVID-19 vaccination among children and younger adults in the United States. Ann Intern Med. (2022). 10.7326/M22-2274 PMC9578536 36191323

[36] https://portal.ct.gov/vaccine-portal/Vaccine-Knowledge-Base/Articles/Kids-Vaccine-Side-Effects?language=en_US.

[37] https://portal.ct.gov/vaccine-portal/Vaccine-Knowledge-Base/Articles/Long-Term-Effects-in-Kids?language=en_US.

[38] Edwards KM Hackell JM, The committee on infectious diseases TCOP, medicine A. Countering vaccine hesitancy. Pediatrics. (2016) 138(3). PMID: 27573088. 10.1542/peds.2016-2146 27573088

[39] Rief W. editor Fear of adverse effects and COVID-19 vaccine hesitancy: recommendations of the treatment expectation expert group. JAMA Health Forum. (2021), American Medical Association. 10.1001/jamahealthforum.2021.0804 36218819

[40] Ruiz JB Bell RA. Parental COVID-19 vaccine hesitancy in the United States. Public Health Rep. (2022) 137(6):1162–9. 10.1177/00333549221114346 35915993 PMC9574308

[41] Yigit M Ozkaya-Parlakay A Senel E. Evaluation of COVID-19 vaccine refusal in parents. Pediatr Infect Dis J. (2021) 40(4):e134–6. 10.1097/INF.0000000000003042 33410650

[42] Tuckerman J Kaufman J Danchin M. Effective approaches to combat vaccine hesitancy. Pediatr Infect Dis J. (2022) 41(5):e243–5. 10.1097/INF.0000000000003499 35213864 PMC8997018

[43] https://www.cdc.gov/vaccines/covid-19/planning/children/resources-promote.html.

[44] https://www.who.int/news-room/spotlight/ten-threats-to-global-health-in-2019.

[45] https://www.nichq.org/insight/economics-creative-paradigm-importance-trust-pediatric-care.

[46] Richard-Eaglin A McFarland ML. Applying cultural intelligence to improve vaccine hesitancy among black, indigenous, and people of color. Nursing Clinics. (2022). 10.1016/j.cnur.2022.04.008 PMC9296256 35985729

[47] Mondal P Sinharoy A Sankoorikal B-J Siddaiah R Mazur L Graff G. The influence of sociodemographic heterogeneity on the perceptions of COVID-19: a countrywide survey study in the USA. Int J Environ Res Public Health. (2021) 18(17):8922. 10.3390/ijerph18178922 34501512 PMC8431068

[48] Shavers VL Lynch CF Burmeister LF. Racial differences in factors that influence the willingness to participate in medical research studies. Ann Epidemiol. (2002) 12(4):248–56. 10.1016/S1047-2797(01)00265-4 11988413

[49] https://www.cmadocs.org/newsroom/news/view/ArticleId/49967/Physicians-urged-to-remain-vigilant-in-recommending-COVID-19-and-flu-vaccines-for-kids.

